# Up-regulated aldo-keto reductase family 1 member B10 in chronic hepatitis C: association with serum alpha-fetoprotein and hepatocellular carcinoma

**DOI:** 10.1111/j.1478-3231.2012.02827.x

**Published:** 2012-06-11

**Authors:** Shunsuke Sato, Takuya Genda, Katsuharu Hirano, Hironori Tsuzura, Yutaka Narita, Yoshio Kanemitsu, Tetsu Kikuchi, Katsuyori Iijima, Ryo Wada, Takafumi Ichida

**Affiliations:** 1 Department of Gastroenterology and Hepatology, Juntendo University Shizuoka HospitalShizuoka, Japan; 2 Department of Pathology, Juntendo University Shizuoka HospitalShizuoka, Japan

**Keywords:** AKR1B10, alpha-fetoprotein, carcinogenesis, chronic hepatitis C, liver, microarray, risk factor

## Abstract

**Background:**

Elevated serum alpha-fetoprotein (AFP) is not only a diagnostic marker for hepatocellular carcinoma (HCC), but is also a risk factor for HCC in chronic hepatitis C patients who do not have HCC.

**Aim:**

The aim was to analyse the hepatic gene expression signature in chronic hepatitis C patients with elevated AFP, who were at high risk for HCC.

**Methods:**

Liver tissue samples from 48 chronic hepatitis C patients were stratified by their serum AFP levels and analysed for gene expression profiles. The association between aldo-keto reductase family 1 member B10 (AKR1B10) expression and serum AFP was confirmed by quantitative real-time reverse transcription polymerase chain reaction (qRT-PCR) and immunohistochemical analyses. A matched case-control study was performed to evaluate the risk of AKR1B10 expression for HCC development.

**Results:**

Distinct hepatic gene expression patterns were demonstrated in patients with elevated AFP (≥10 ng/mL) and normal AFP (<10 ng/mL). Of the 627 differently expressed genes, the most abundantly expressed gene in patients with elevated AFP was AKR1B10 (fold change, 26.2; *P* < 0.001), which was originally isolated as an overexpressed gene in human HCC. The qRT-PCR and immunohistochemical studies confirmed a proportional correlation between AKR1B10 expression and serum AFP. A matched case-control study identified that AKR1B10 up-regulation (>6%) was an independent risk factor for HCC development (hazard ratio, 21.4; *P* = 0.001).

**Conclusion:**

AKR1B10 was up-regulated in association with serum AFP, and was an independent risk factor for HCC in chronic hepatitis C patients, suggesting its possible involvement at a very early stage of hepatocarcinogenesis.

Hepatocellular carcinoma (HCC) is the fifth most common cancer and the third most common cause of cancer-related death worldwide 1. Approximately 70–90% of patients with HCC have an established background of chronic liver disease and cirrhosis 1. Persistent infection with the hepatitis C virus (HCV) is one of the major causes of chronic liver disease leading to the development of HCC. Persistent HCV infection is responsible for 27–75% of the HCC cases in Europe and the United States and >80% of the HCC cases in Japan 2, 3. The annual incidence of HCC development is 2–8% in cirrhotic patients with chronic HCV infection 4, 5. Persons with anti-HCV positivity were shown to have a 20-fold increased risk of developing HCC in comparison with those who were negative for anti-HCV 6. Thus, the carcinogenic role of persistent HCV infection appears to be significant. However, the molecular mechanism of HCV-related hepatocarcinogenesis is not completely understood, particularly in its early stages.

Alpha-fetoprotein (AFP) is the most thoroughly characterized carcinofetal gene product and its usefulness in the surveillance and diagnosis of HCC is well established. On the other hand, AFP elevation is recognized not only in patients with HCC but also in patients with chronic viral hepatitis or cirrhosis, who have no evidence of HCC. AFP elevation was observed in over 15% of patients with chronic hepatitis C in the absence of HCC 7. In addition, several studies have indicated that AFP elevation is a significant predictor of HCC development 8–10. Recent reports reveal that the estimated HCC risk in patients with elevated AFP is over three-fold higher than that in patients with normal AFP 11, 12. These observations suggest that the molecular alterations associated with the very early stages of hepatocarcinogenesis have occurred in the livers of patients with chronic hepatitis C with AFP elevation. In this study, we attempted to identify a specific gene expression signature by performing microarray analysis on the livers of patients with chronic hepatitis C and AFP elevation, which is considered high risk for development of HCC.

## Materials and methods

### Patients and sample preparation

Liver tissues were obtained from patients with chronic hepatitis C via percutaneous liver biopsy at Juntendo University Shizuoka Hospital (Shizuoka, Japan). Chronic hepatitis C was diagnosed on the basis of anti-HCV status and detectable serum HCV RNA. All the patients were negative for hepatitis B surface antigen and showed no evidence of HCC on ultrasonography or computed tomography before biopsy. Histological grading and staging were performed according to the Metavir classification system 13. Blood chemistry values for the following factors were determined: complete blood count, alanine aminotransferase (ALT), γ-glutamyl transpeptidase (γGTP) and AFP. In this study, we defined elevated AFP as a value of ≥10 ng/mL, according to previous reports 10, 12. Normal liver tissues without unusual histological features were obtained from nontumoral parts of livers complicated with colorectal metastasis and were used as control liver tissues.

This study was approved by the Ethical Committee of Juntendo University Shizuoka Hospital in accordance with the Helsinki Declaration, and written informed consent was obtained from all patients.

### RNA preparation and microarray hybridization

Total RNA was isolated using the RNeasy Mini Kit (Qiagen, Hilden, Germany) and evaluated by the Agilent 2100 Bioanalyzer (Agilent Technologies, Palo Alto, CA, USA). Only high-quality RNA with RNA integrity numbers greater than 7.0 was used for experiments. The Agilent Whole Human Genome Microarray (design ID, 014850), which contains 44,000 60-mer oligonucleotide probes representing 41,000 genes and transcripts, was used to generate gene expression profiles. Total RNA (250 ng) was labelled and hybridized according to the One-Color Microarray-Based Gene Expression Analysis protocol ver.5.7. Hybridization signals were detected using the DNA microarray scanner G2505B (Agilent Technologies).

### Microarray data analysis

The intensity values of each scanned feature were quantified using Agilent feature extraction software (v10.7.1.1, Agilent Technologies). We only used features that were flagged as no errors (present flags) and excluded features that were not positive, not significant, not uniform, not above background, saturated and population outliers (marginal and absent flags). Normalization was performed using GeneSpring GX 10.0.2 software (Agilent Technologies) (per chip, normalization to 75 percentile shift; per gene, normalization to median of all samples). Data filtration resulted in 30,150 probes as a valid expressed gene set in which at least 24 of the 48 samples had present flags for further analysis. Raw microarray data were deposited in Gene Expression Omnibus (GSE32221) and are available to the public.

The altered transcripts were quantified using the comparative method. Statistical analysis between groups was performed by the unpaired unequal variance Welch's *t*-test, and multiple testing corrections were performed by determining the false discovery rate (FDR) using GeneSpring software. Altered gene expression was considered significant if the transcript had an FDR corrected to *P* ≤ 0.05 and ≥ 2-fold change in signal intensity. Principal component analysis (PCA), hierarchical clustering analysis (HCA) and gene ontology (GO) analysis were performed using GeneSpring software.

### Quantitative real-time RT-PCR for RNA quantification (qRT-PCR)

TaqMan real-time RT-PCR was performed to quantify the relative expression levels of aldo-keto reductase family 1, member B10 (AKR1B10) (assay ID, Hs01546975_gH) and the beta-actin housekeeping gene (assay ID, Hs99999903_m1) (Applied Biosystems, Foster City, CA, USA). cDNA was synthesized from 1 μg total RNA by using Superscript reverse transcriptase (Invitrogen, Carlsbad, CA, USA) with oligo dT primers, according to the manufacturer's instructions. Specific mRNA was quantified with a LightCycler 480 (Roche, Mannheim, Germany) by using 2 × Premix Ex Taq (TaKaRa BIO, Shiga, Japan). All PCR reactions were performed in triplicate. The relative expression of AKR1B10 was calculated using the comparative cycle threshold (delta C_T_) method as described previously 14.

### Immunohistochemistry

AKR1B10 immunohistochemical analysis was performed as described previously with some modifications 14. In brief, deparaffinized and rehydrated sections were processed by heat-induced antigen retrieval in 0.1 M citrate buffer at pH 6.0. After blocking the endogenous peroxidase activity, the sections were incubated with a mouse monoclonal antibody against AKR1B10 (Ab 57547; Abcam, Cambridge, UK) with a 1:100 dilution at room temperature, followed by incubation with biotinylated secondary antibody (Ventana iVIEW DAB Universal Kit; Ventana Medical Systems Inc., Tucson, AZ, USA). Staining was visualized using 3'-3-diaminobenzidine tetrahydrochloride and hematoxylin counterstain. Negative controls were performed by replacing the primary antibody with mouse immunoglobin (Sigma–Aldrich Biochemicals, St. Louis, MO, USA). AKR1B10 immunostaining was based on positive cytoplasmic staining and was quantitatively assessed as the average percentage of AKR1B10 positive areas in two independent fields at 100 × magnification by using Lumina Vision 2.4 Bio-imaging software (Mitani Corporation, Tokyo, Japan).

### Matched case-control study

From January 2005 to April 2010, 278 consecutive patients with chronic hepatitis C underwent liver biopsy followed by periodic HCC surveillance by using ultrasonography or computed tomography at least every 4 months. All the patients had a minimum follow-up duration of 12 months after liver biopsy. HCC was diagnosed by histological examination and/or triphasic computerized tomography, in which hyperattenuation in the arterial phase with washout in the late phase is pathognomonic for HCC 15. For each patient who developed HCC, two control patients who matched the HCC patient in terms of gender, age (within 5 years) and histological fibrosis stage were randomly selected from the patients who did not develop HCC during the follow-up period.

### Statistical analysis

Statistical analysis was performed using the Mann–Whitney *U*-test for comparison of continuous variables between groups and the corrected Chi-squared method for comparison of qualitative data. Univariate and multivariate Cox proportional hazard models were used to assess factors that were significantly associated with HCC development. The hazard ratio and 95% confidence interval (CI) were also calculated. All statistical analyses were performed using IBM spss 13.0 (IBM SPSS, Chicago, IL, USA). *P* < 0.05 was considered statistically significant.

## Results

### Gene expression profiling by microarray analysis

The baseline characteristics of the 48 patients who were enrolled in the microarray analysis are summarized in Table 1. Fifteen (31%) of the 48 patients showed elevation of serum AFP (≥10.0 ng/mL). There were no significant differences between patients with elevated AFP and those with normal AFP in terms of age, gender, body mass index or histological grade of necroinflammation. Patients with elevated AFP showed higher serum ALT, lower platelet count and further progression of liver fibrosis compared to those with normal AFP.

**Table 1:** Baseline characteristics of patients enrolled in the microarray analysis

Variables	All patients	Patients with normal AFP (<10 ng/mL)	Patients with elevated AFP (≥10 ng/mL)	*P* value†
Number	*N* = 48	*N* = 33	*N* = 15	
Age (years)*	59.5 (32–78)	57.0 (32–78)	65.0 (44–78)	0.142
Gender (male/female)	28/20	21/12	7/8	0.349
Body mass index (kg/cm^2^)*	22.7 (17.5–30.4)	22.8 (18.6–30.4)	22.5 (17.5–29.0)	0.755
ALT (IU/L)*	61.5 (10–290)	39.0 (10–270)	162.0 (54–290)	<0.001
γGTP (IU/L)*	38.5 (9–540)	28.0 (9–119)	16 (28–540)	<0.001
Platelet count (10^4^/μL)*	18.8 (5.2–35.4)	20.5 (8.3–35.4)	14.8 (5.2–22.4)	<0.001
AFP (ng/μL)*	5.0 (2–120)	4.0 (2–9)	19.0 (10–120)	<0.001
Staging (F0-F2/F3-F4)	41/7	33/0	8/7	<0.001
Grading (A0-A1/A2-A3)	12/26	10/23	2/13	0.292

ALT, alanine aminotransferase; AFP, alpha-fetoprotein; γGTP, γ-glutamyl transpeptidase.

* Data are expressed as median (range).

† The *P* value was determined using the Mann–Whitney *U*-test, the Chi-square test, and Fisher's exact probability test.

**Fig. 1:**
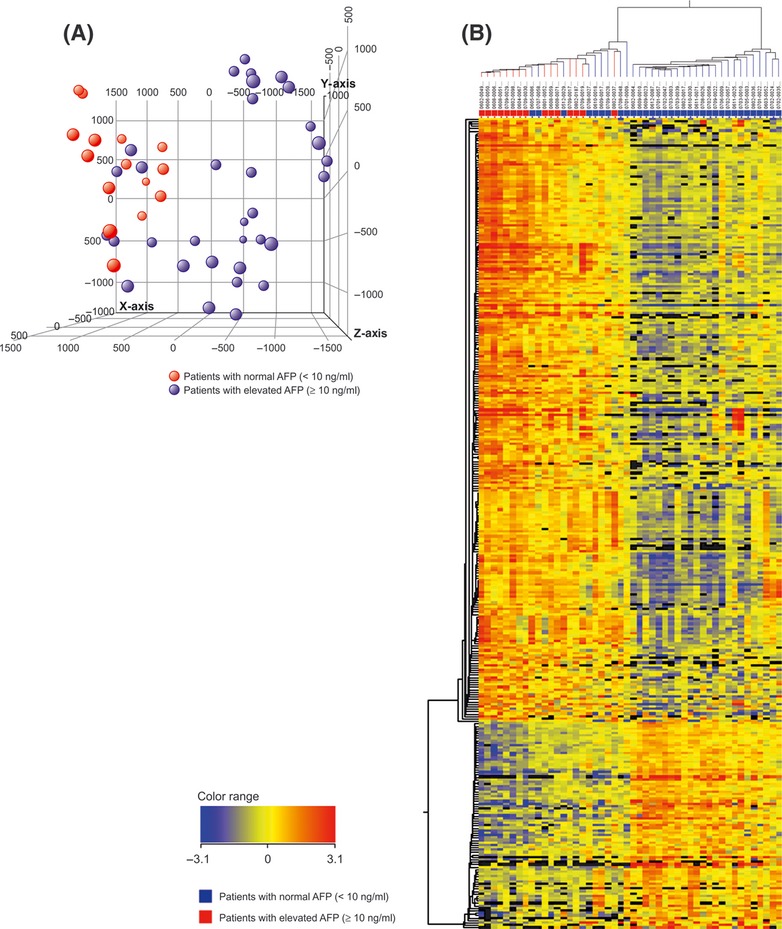
Analysis of gene expression data. (A) Principal component analysis for the 627 differentially expressed genes between the patients with normal alpha-fetoprotein (AFP) (*N* = 33, *blue dots*) and elevated AFP (*N* = 15, *red dots*). (B) Hierarchical clustering for the 627 differentially expressed genes between the patients with normal AFP (*N* = 33, *blue squares*) and elevated AFP (*N* = 15, *red squares*). Red and blue cells indicate the ratio of each expression level above and below the median respectively.

Among the 30,150 valid genes, 627 were identified as differentially expressed genes with a minimal fold change of 2.0. Using these 627 genes, PCA and HCA were used to successfully distinguish samples according to their AFP status. Patients with elevated AFP were divided from the cluster of those with normal AFP in scatter spot graphics of PCA (Fig. 1A). HCA resulted in the formation of two main clusters, one comprising 27 of the 33 (81.8%) patients with normal AFP and the other comprising 14 of the 15 (93.3%) patients with elevated AFP (Fig. 1B). Classification of these 627 genes according to GO function demonstrated that patients with elevated AFP showed up-regulation of genes associated with the GO terms ‘immune response,’ ‘DNA replication,’ ‘biological adhesion’ and ‘cell adhesion’ (Table S1).

### Up-regulation of AKR1B10 in patients with elevated AFP

**Fig. 2:**
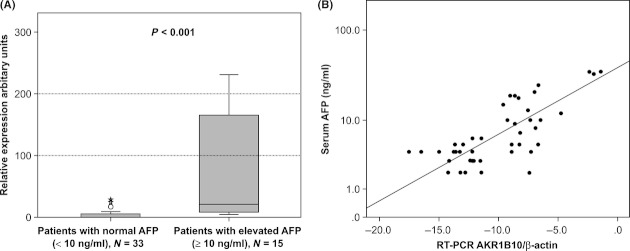
Quantitative real-time RT-PCR analysis. (A) Comparison of AKR1B10 mRNA expression between the patients with normal alpha-fetoprotein (AFP) and the patients with elevated AFP (Mann–Whitney *U*-test, *P* < 0.001). (B) Regression of AKR1B10 mRNA and serum AFP (*N* = 48, *R*^2^ = 0.326, *P* < 0.001).

The top 20 differentially expressed genes are shown in Table 2. Among them, AKR1B10 was the most abundantly up-regulated gene in patients with elevated AFP (26.1-fold change, *P* < 0.001). The microarray data were validated by qRT-PCR. The expression of AKR1B10 mRNA was significantly higher in patients with elevated AFP than in patients with normal AFP: median 22.3 arbitrary units vs. 0.58 arbitrary units respectively (*P* < 0.001) (Fig. 2A). In addition, regression analysis showed a significant correlation between AKR1B10 mRNA and serum AFP (Fig. 2B).

**Table 2:** Differentially expressed genes in patients with elevated alpha-fetoprotein by fold change ranking (Top20)

Symbol	Title	Up or down	*P* value	Fold change
AKR1B10	Aldo-keto reductase family 1, member B10	Up	9.19E-07	26.2
KRT23	Keratin 23	Up	4.17E-07	22.6
GPC3	Glypican 3	Up	3.54E-09	12.3
FAM3B	Family with sequence similarity 3, member B	Up	1.67E-06	10.5
HKDC1	Hexokinase domain containing 1	Up	3.89E-07	8.8
EpCAM	Epithelial cell adhesion molecule (TACSTD1)	Up	3.07E-06	7.6
DHRS2	Dehydrogenase/reductase (SDR family) member 2	Down	2.39E-04	7.0
OSTbeta	Organic solute transporter beta	Up	3.14E-06	7.0
NRXN3	Neurexin 3	Up	1.40E-04	6.7
CHI3L1	Chitinase 3-like 1	Up	2.92E-06	6.5
TMEM125	Transmembrane protein 125	Up	9.21E-06	6.3
KCNN2	Potassium intermediate/small conductance calcium-activated channel, subfamily N	Down	5.68E-04	6.2
PDZK1IP1	PDZK1 interacting protein 1	Up	1.39E-05	6.1
RAB25	RAB25, member RAS oncogene family	Up	1.73E-05	6.0
DIO3OS	Deiodinase, iodothyronine, type 3 opposite strand	Down	1.87E-06	5.6
LYPD1	LY6/PLAUR domain containing 1	Up	3.97E-05	5.5
STMN2	Stathmin-like 2	Up	5.53E-04	5.1
LOXL4	Lysyl oxidase-like 4	Up	3.91E-06	5.1
KLHL29	Kelch-like protein 29	Up	3.68E-08	5.0
TMC4	Transmembrane channel-like 4	Up	3.64E-06	5.0

**Fig. 3:**
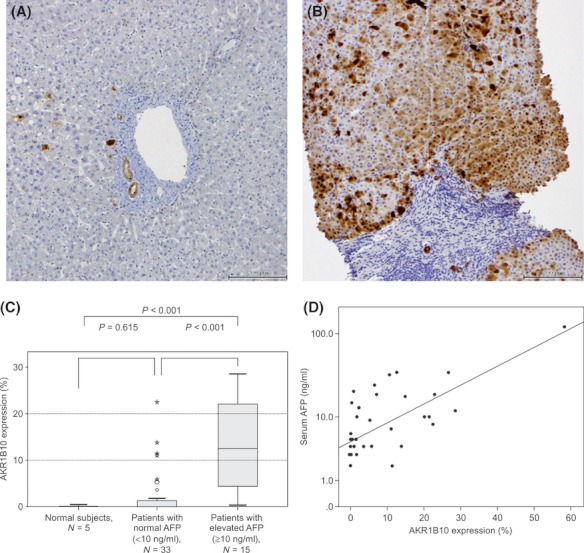
AKR1B10 immnohistochemical analysis. (A) Representative immunohistochemical staining of normal control liver tissue. Bile duct epithelium served as the positive control. (B) Representative immunohistochemical staining of liver tissue with chronic hepatitis C (magnification × 100). (C) Quantification of AKR1B10 immunoreactivity (Mann–Whitney *U*-test). (D) Regression of AKR1B10 immunoreactivity and serum alpha-fetoprotein in patients with chronic hepatitis C (*N* = 48, *R*^2^ = 0.613, *P* < 0.001).

To further analyse AKR1B10 expression, immunohistochemical analysis was performed using a monoclonal antibody against AKR1B10. In the normal control liver tissues, immunoreactivity was mainly observed in bile duct cells, whereas reactivity was rarely observed in the cytoplasm of these hepatocytes (Fig. 3A). In contrast, hepatocytes in livers with chronic hepatitis C showed prominent AKR1B10 immunoreactivity in the cytoplasm, or in the cytoplasm and nucleus (Fig. 3B). The AKR1B10-positive hepatocytes were mostly localized in the periportal zone. Quantitative image analysis revealed that the median percentage of the AKR1B10 positive area was 0.16, 0.16 and 12.57% in control subjects, patients with normal AFP and patients with elevated AFP respectively. The AKR1B10 positive areas were significantly greater in patients with elevated AFP than in control subjects and patients with normal AFP (*P* < 0.001) (Fig. 3C). Although several patients with normal AFP exhibited elevated AKR1B10 immunoreactivity, these differences were not statistically significant between control subjects and patients with normal AFP. Similar to the qRT-PCR results, regression analysis showed a significant correlation between AKR1B10 immunoreactivity and serum AFP (Fig. 3D).

### AKR1B10 expression and risk of HCC

**Fig. 4:**
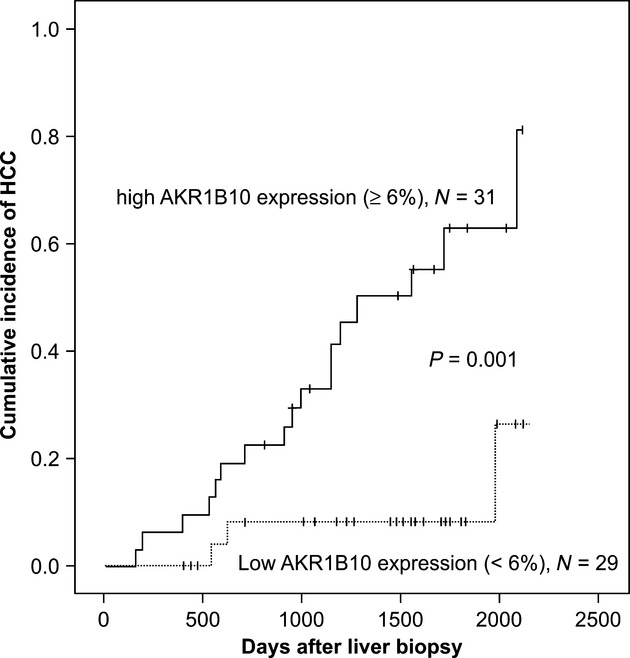
Cumulative incidence of hepatocellular carcinoma development according to AKR1B10 expression (log-rank test, *P* = 0.001).

A matched case-control study was performed to evaluate the risk of AKR1B10 expression for HCC development. During the follow-up period after liver biopsy, 20 of 278 chronic hepatitis C patients developed HCC. A comparison of patient characteristics between HCC and control cases is shown in Table 3. According to the case-match design, age, gender and fibrosis stage were similar in both groups, but serum ALT and AFP were significantly higher in HCC cases than in control cases. Immunohistochemical analysis demonstrated that AKR1B10 expression was significantly higher in HCC cases than in control cases (17.7% vs. 1.2%, *P* = 0.001). Table 4 shows the Cox proportional hazard ratios for HCC development estimated with univariate and multivariate models. Univariate analysis identified four factors that were significantly associated with HCC development: ALT (≥90 IU/L, *P* = 0.037), platelet count (≤10 × 10^4^/μL, *P* = 0.005), AFP (≥13 ng/mL, *P* = 0.012) and AKR1B10 expression (≥6%, *P* = 0.006). Multivariate analysis identified two independent factors that were significantly associated with HCC development: expression of AKR1B10 (hazard ratio, 21.45; *P* = 0.023) and platelet count (hazard ratio, 17.46; *P* = 0.029). Kaplan-Meier plot analysis and the log-rank test showed a significant difference in cumulative incidence of HCC development between cases with high (≥6%) or low (<6%) expression of AKR1B10 (Fig. 4).

**Table 3:** Baseline characteristics of patients enrolled in the matched case-control study

Variables	HCC cases (*N* = 20)	Control cases (*N* = 40)	*P* value†
Gender (male/female)	14/6	28/12	Matched
Age (years)*	65 (44–79)	66 (44–80)	Matched
Staging (F1/F2/F3/F4)	3/2/11/4	6/4/22/8	Matched
Body mass index (kg/m^2^)*	23.0 (17.5–31.3)	23.1 (17.3–28.6)	0.878
ALT (IU/L)*	97 (32–489)	53 (17–699)	0.017
γGTP (IU/L)*	79 (24–161)	45 (13–375)	0.063
Platelet count (×10^4^/μL)	9.9 (5.1–17.3)	15.4 (9.1–24.4)	<0.001
AFP (ng/mL)*	20 (3–142)	6 (1–576)	0.007
Interferon therapy (Yes/No)	16/4	37/3	0.208
Viral clearance (Yes/No)	6/14	21/19	0.099
AKR1B10 expression (%)*	17.7 (0–66.6)	1.2 (0–41.0)	0.001
Follow-up duration (days)*	920 (164–2079)	1534 (406–2118)	0.004

ALT, alanine aminotransferase; AFP, alpha-fetoprotein; γGTP, γ-glutamyl transpeptidase; HCC, hepatocellular carcinoma.

* Data are shown as median (range).

† The *P* value was determined using the Mann–Whitney *U*-test, the chi-square test and Fisher's exact probability test.

**Table 4:** Univariate and multivariate analysis for predictors of hepatocellular carcinoma development

		Univariate analysis		Multivariate analysis	
			
Variable	Category	Hazard ratio (95% CI)	*P* value	Hazard ratio (95% CI)	*P* value*
Body mass index (kg/m^2^)	0: <25.2	1	0.332		
	1: ≥25.2	1.75 (0.57–5.42)			
ALT (IU/L)	0: <90	1	0.037		
	1: ≥90	3.53 (1.08–11.56)			
γGTP (IU/L)	0: <65	1	0.061		
	1: ≥65	3.20 (0.95–10.81)			
Platelet count (×10^4^/μL)	0: ≥10.0	1	0.005	1	0.029
	1: <10.0	19.08 (2.44–149.10)		17.46 (1.34–226.81)	
AFP (ng/mL)	0: <13	1	0.012		
	1: ≥13	5.26 (1.43–19.31)			
AKR1B10 expression (%)	0: <6	1	0.006	1	0.023
	1: ≥6	17.79 (2.29–138.33)		21.45 (1.54–310.86)	
Interferon therapy	0: Yes	1	0.149		
	1: No	5.26 (0.55–50.02)			
Viral clearance	0: Yes	1	0.120	1	0.057
	1: No	2.41 (0.80–7.32)		10.34 (0.93–114.40)		

ALT, alanine aminotransferase; AFP, alpha-fetoprotein; γGTP, γ-glutamyl transpeptidase.

* The *P* value was determined using the Cox proportional hazard model.

## Discussion

Although AFP is widely used in the surveillance and diagnosis of HCC, the AFP level is sometimes elevated in chronic liver disease patients who have no evidence of HCC. Measurement of AFP is clinically important despite its lack of specificity because elevated serum AFP in benign liver disease is a significant predictor of HCC 7–12. Therefore, livers with elevated AFP are at a higher carcinogenic risk than those with normal AFP. In this study, to precisely investigate the molecular alteration in the very early stages of hepatocarcinogenesis, we used a cDNA microarray-based strategy to compare the gene expression profiles of chronic hepatitis C patients who were stratified according to their AFP levels. The PCA and HCA of our microarray data demonstrated a clear difference in the intrahepatic molecular signatures between patients with elevated AFP and those with normal AFP. GO analysis revealed that genes up-regulated in patients with elevated AFP were enriched in the GO terms ‘immune response’ (e.g., *IFI6*, *TREM2*, *ISG15* and *CXCL10*) and ‘DNA replication’ (e.g., *CDC6* and *CDC45L*). Serum AFP is thought to be increased both by hepatocyte injury and up-regulation of its turnover, because it is correlated with serum ALT and histological necroinflammation in patients with chronic hepatitis C 7. The hepatic gene signatures in patients with elevated AFP observed in our GO analysis substantiate this theory.

Among the 627 differentially expressed genes, AKR1B10 was the most highly up-regulated gene in patients with elevated AFP. AKR1B10 is a member of the AKR superfamily and was originally isolated as a gene whose expression was increased in human HCC. Previous studies reported faint AKR1B10 expression in the normal liver and frequent over-expression in human HCC 14, 16, 17. However, it remained unclear whether AKR1B10 expression was altered in patients with chronic liver disease, particularly in that associated with chronic hepatitis C. In this study, AKR1B10 expression was significantly up-regulated in patients with chronic hepatitis C and elevated AFP compared to normal liver control subjects or patients with chronic hepatitis C and normal AFP. More importantly, regression analysis revealed a significant correlation between AKR1B10 expression and serum AFP. To our knowledge, this is the first report to show up-regulation of intrahepatic AKR1B10 expression in association with serum AFP in patients with chronic hepatitis C. The mechanism by which intrahepatic AKR1B10 was up-regulated in chronic hepatitis C remains largely unknown. Although previous studies reported that AKR1B10 expression was regulated by the transcription factors AP-1 and Nrf-2 18, 19, changes of AP-1 and Nrf-2 gene expression were not observed in our microarray analysis (data not shown). Further studies are warranted to better understand the mechanism of AKR1B10 regulation in chronic hepatitis C.

It is not clear why AKR1B10 expression is correlated with AFP. Aldo-keto reductase enzymes are NAD(P)H-dependent oxidoreductases that catalyse the reduction of carbonyl compounds, and various physiological substrates have been proposed for many AKR enzymes. Recently, AKR1B10 was shown to have a high catalytic efficiency for the reduction of all-*trans*-, 9-*cis*- and 13-*cis*-retinals to their corresponding retinols *in vitro* and *in vivo* 20, 21. Conversion of retinals to retinols via AKR1B10 can deprive retinoic acid receptors of their ligands, and can presumably inhibit the retinoic acid signalling pathway 22. Retinoic acid is thought to be essential for the maintenance of normal epithelial differentiation. Retinoic acid depletion causes cell proliferation and loss of differentiation, thereby inducing preneoplastic phenotypes in normal epithelium 23–25. On the other hand, retinoic acid exposure inhibits proliferation of normal and transformed cells *in vitro* 26, 27, and dietary retinoic acid reduced the development of premalignant and malignant lesions in a chemically induced mouse carcinogenesis model 28. In human HCC, oral administration of acyclic retinoids is reported to prevent HCC 29. Collectively, these data indicate that up-regulation of AKR1B10 is linked to the depletion of retinoic acid levels, subsequent loss of differentiation and induction of the carcinofetal phenotype in hepatocytes, resulting in elevated serum AFP. Interestingly, our microarray analysis identified that dehydrogenase/reductase member 2 (DHRS2) was the most down-regulated gene in patients with elevated AFP. DHRS2 was previously known as a nuclear protein Hep27 and functions in inhibition of cell proliferation through p53 stabilization 30. Therefore, DHRS2 down-regulation is likely to result in hepatocyte proliferation. Taken together, not only AKR1B10 up-regulation but also alteration of other molecules, such as DHRS2, might be involved in the mechanisms of serum AFP elevation.

In the matched case-control study, AKR1B10 expression and platelet count were identified as independent predictors of HCC development. In particular, a ≥6% up-regulation of AKR1B10 was associated with a ≥21-fold relative risk. Many studies have shown AKR1B10 up-regulation in several types of cancers, including recent reports of HCC 14, 31, 32, as well as in precancerous conditions, such as squamous metaplasia and Barrett's oesophagus 33, 34. Furthermore, several reports have shown that down-regulation of AKR1B10 by using small interfering RNA inhibited cancer cell proliferation both *in vitro* and *in vivo* 31, 35. Thus, the involvement of AKR1B10 in carcinogenesis is intriguing. Collectively, our data and these studies indicate that AKR1B10 is not only a useful predictive marker of HCC but also might play an important role in hepatocarcinogenesis, particularly in the very early stages. Consistently, previous studies reported that AKR1B10 up-regulation was mainly observed in early-stage HCC with well differentiation, and rarely observed in advanced stage HCC with poor differentiation 32, 36, indicating AKR1B10 up-regulation is an early event in the process of hepatocarcinogenesis.

In conclusion, this study demonstrated that intrahepatic AKR1B10 expression was up-regulated in association with AFP and significantly reflected the risk of HCC in patients with chronic hepatitis C. AKR1B10 is not only a clinically useful predictive marker for HCC development but may also hold the key to elucidating the mechanism of the very early stages of hepatocarcinogenesis. Our findings might reveal a new insight into the molecular mechanism of hepatocarcinogenesis and provide a novel therapeutic target for the prevention of HCC.
